# ﻿*Ormosianeillii* (Fabaceae), a remarkable new tree species from the Cordillera del Cóndor plateaus in Ecuador

**DOI:** 10.3897/phytokeys.256.147923

**Published:** 2025-05-05

**Authors:** Juan Ernesto Guevara-Andino, John L. Clark, Daniel Navas-Muñoz

**Affiliations:** 1 Grupo de Investigación en Ecología y Evolución en los Trópicos-EETrop- Universidad de las Américas, Quito 170124, Ecuador Universidad de las Américas Quito Ecuador; 2 Marie Selby Botanical Gardens, 1534 Mound Street, Sarasota, FL 34236, USA Marie Selby Botanical Gardens Sarasota United States of America; 3 Instituto Nacional de Biodiversidad, Herbario Nacional del Ecuador QCNE, Quito, Ecuador Instituto Nacional de Biodiversidad, Herbario Nacional del Ecuador QCNE Quito Ecuador

**Keywords:** Endemism, Fabaceae, Papilionoideae, tepui-like formation, white sands

## Abstract

A new species, *Ormosianeillii* (Fabaceae), is described and illustrated from the tepui-like formations of the Cordillera del Cóndor Region in south-eastern Ecuador. Morphological similarities with other species of *Ormosia* are examined and discussed. Based on IUCN guidelines, a preliminary conservation status of Endangered (EN) is recommended.

## ﻿Introduction

*Ormosia* Jackson is a papilionoid legume genus (Fabaceae, Papilionoideae) with tropical and subtropical distribution, comprising more than 150 species from treelets and shrubs to canopy trees (Rudd 1965; Cardoso and de Queiroz. 2010; Cardoso et al. 2012; Cardoso et al. 2014; Torke et al. 2022). The geographical distribution of *Ormosia* constitutes a clear example of an Asian-American tropical disjunction pattern and recent molecular evidence suggests a Palaeotropical origin in Asia (Torke et al. 2022). Historically, *Ormosia* has been classified within the polyphyletic tribe Sophoreae, but molecular evidence suggests that *Ormosia*, together with the genera *Clathrotropis* Harms, *Panurea* Spruce ex Benth. and *Spirotropis* Tul., can be grouped in the so called “Ormosieae clade” (Cardoso et al. 2013, 2017). More recent phylogenetic studies suggest that *Clathrotropis*, *Panurea* and *Spirotropis* share a recent common ancestor and are referred to as the Clathrotropisoid clade (Torke et al. 2022), which does not include *Ormosia* (Lavin et al. 2005; Cardoso et al. 2013; Torke et al. 2022). *Ormosia* is characterised the following features: imbricate calyx lobes; ten free stamens; an incurved style; a terminal or oblique, often bilobed stigma; and seeds with hard testa, which are frequently red, black or bicoloured red and black (Rudd 1965; Cardoso and de Queiroz 2010). In the most comprehensive taxonomic revision of Neotropical *Ormosia*, Rudd (1965) defined the following three sections based mostly on the morphology of seed characters: sect. Ormosia, sect. Macrocarpae Ducke and sect. Unicolores Amsh. Based on Rudd’s classification, sect. Unicolores is characterised by the unicoloured seeds (e.g. red, black or yellowish, but sometimes spots of black). In Ecuador, eight species of *Ormosia* have been recorded, occurring in Amazonian lowlands below 500 m a.s.l., Andean foothills up to 1000 m a.s.l. and in the Chocó Biogeographic Region in the north-western Andes of Ecuador below 500 m a.s.l. (Ulloa-Ulloa et al. 2021. Even though Ecuadorian Amazonia has been botanically well explored throughout the last 35 years, there are some regions that remain relatively unexplored or biodiversity “darkspots” with several undocumented and undescribed tree species (see Ondo et al. (2024)). The Cordillera del Cóndor, despite recent large-scale floristic inventories and botanical exploration, remains poorly known.

The Cordillera del Cóndor, a sub-Andean range situated between the Andes and the Amazon, spans about 1.1 million hectares and stretches 150 km along the Ecuador-Peru border (Neill 2005). Known as a biodiversity hotspot of endemism for both plants and animals, the northern part of this region shares floristic similarities with the Guiana Shield (Neill 2005; Ulloa Ulloa and Neill 2006). Recent discoveries on the Cordillera’s sandstone plateaus have revealed several new species (Ulloa Ulloa et al. 2012; Prance 2013; Guevara-Andino and Fernández-Alonso 2018; Fernández-Fernández et al. 2020; Clark and Neill 2023) and *Incadendron* K. Wurdack & Farfan, a recently described monotypic genus (Wurdack and Farfan-Ríos 2017). Our large-scale inventories on the white sand forests have also led to the identification of a previously unknown species of *Ormosia* with a remarkable dark purple to black corolla that is only known from the pre-montane white sand forests of this region.

## ﻿Materials and methods

We have performed floristic inventories and botanical collections in the Cordillera del Cóndor since 2017. During one of our floristic inventories, we collected specimens that represent an undescribed species in the genus *Ormosia*. We describe this new species, based on an analysis of morphological characters from material deposited in the following Herbaria: Herbario Nacional del Ecuador (QCNE), Herbario Amazónico del Ecuador (ECUAMZ), Marie Selby Botanical Gardens (SEL) and the Missouri Botanical Garden (MO). We also compared the new species with images of type specimens deposited in JStor Plants (https://plants.jstor.org) and reviewed voucher specimens in the virtual herbaria of the Field Museum (F), the New York Botanical Garden (NY), the Herbario Nacional de Colombia (COL) and the Herbário do Instituto Nacional de Pesquisas da Amazônia (INPA; herbarium abbreviations follow Thiers (2025)). In this work, we used the terminology and classification from Rudd (1965). Earlier works have demonstrated that a single diagnostic character, the position of the calyx in relation to the bud, is the only consistent diagnostic character for the circumscription of taxa to subgenus (Rudd 1965; Cardoso and de Queiroz 2010).

The AOO and EOO for the preliminary IUCN assessment were determined using the software package conR in the R statistical software (Dauby et al. 2017). To test the effects of deforestation, we used the most updated data on deforestation for Ecuador from the online platform Mapa Interactivo (Ministerio del Ambiente, Agua y Transición Ecológica del Ecuador 2024). We used ecosystem layers for the Cordillera del Cóndor Region and deforestation maps for this area between 1990 and 2022. Habitat reduction was then estimated combining deforestation on ecosystems with the Extent of Occurrence (EOO) and Area of Occupancy (AOO) using the clip tool in the ArcGis software (ESRI 2011). The clip tool overlays a range size map (EOO and AOO) with a boundary layer or layers, in this case, the deforestation scenarios from 1990 to 2022 and the ecosystem maps. Then, we defined the species’ potential habitat to estimate habitat loss as the area outside the combined boundary of ecosystems and deforestation maps.

## ﻿Taxonomic treatment

### ﻿ Ormosia neillii

Taxon classificationPlantaeFabalesFabaceae

J.L.Clark & J.E.Guevara sp. nov.

0531E23F-3FD0-5CC6-BFA3-BAD28D4AE41B

urn:lsid:ipni.org:names:77361207-1

Figs 1 , 2 , 3 , 4

#### Diagnosis.

*Ormosianeillii* is morphologically similar to *O.cuatrecasasii*, but it can be differentiated by suborbicular to ovate glabrescent fruit with strongly cuspidate apex, smaller leaves (5–14.5 cm long vs. 10–30 cm long), smaller fruits (3.5–6 cm long vs. 5–10 cm long), larger calyx tube (10–15 mm long vs. 6–7 mm long) and uniformly light red to dark red seeds vs. bicolored seeds.

**Figure 1.**
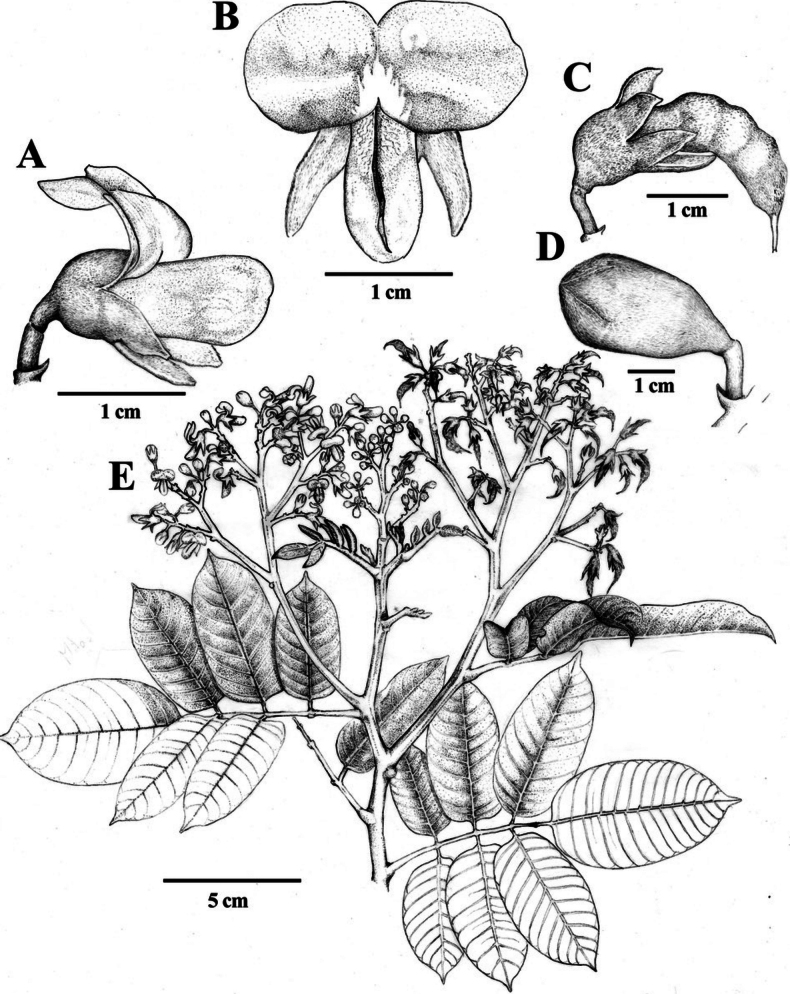
Illustration of *Ormosianeillii* J.L. Clark & J.E. Guevara **A** lateral view of flower **B** front view of corolla **C** fruit **D** immature flower **E** branch with mature fruits and flowers. Illustration by Efrén Merino-Santi.

#### Type.

**Ecuador** • Zamora Chinchipe: Cantón Nangaritza, Parroquia Zumi, Cordillera del Cóndor, western side of tepui (bloque #2) that overlooks Rio Nangaritza, directly east of Cabañas Yankuam, north of Reserva Natural Maycú and located in Área de Conservación Atasmo, 0.25-hectare plot with the Lawrenceville School field course, 8 Mar 2018 (fl, fr), 04°15'13.8"S, 78°38'11.6"W, *John L. Clark & David A. Neill 15775* (holotype: SEL; isotypes: ECUAMZ, MO, NY, US).

#### Description.

Tree 5–10 (15) m tall; trunk with outer bark brown-reddish covered by dark purple lenticels, inner bark reddish with longitudinal white stripes, young branchlets striate, petiole, leaf rachis and pulvinules densely tomentose, with flexuous, appressed, golden and ferruginous hairs or, rarely, glabrescent when in fruit. Stipules absent. Leaves (9.9–)11–26.5 cm long, imparipinnate, 3–7-foliolate; pulvinus 6.4–7 × 2–5 mm, terete; petiole (1.7–) 2–4.5 cm long; rachis (2.5–)5.5–16.5 cm long, interfoliolar segments 1–4 cm long; stipels absent; leaflets opposite, the pulvinules 4.3–4.5 × 1–2 mm, terete, the blades (5–)8–14.5 × (2–)3.5–7 cm, chartaceous, oblong-elliptic to broadly elliptic, basally truncate, apically acute and the margin slightly revolute, pubescent on the abaxial surface, the indumentum of adpressed yellowish hairs, the mid-vein abaxially prominent, the secondary veins in 10–12 pairs, eucamptodromous, well-raised abaxially, mostly 8–10 mm apart, arcuate, forming angles of 60°–75° with the mid-vein, the tertiary veins reticulate, but inconspicuous. Panicle 12–15 cm long, terminal compact, composed by 3–10 racemes of 3–6.5 cm long; axes, bracts, bracteoles and pedicels densely tomentose, covered by erect, flexuous, yellowish hairs; bracts absent, pedicel 1.2–1.5 mm long, bearing one minute bracteole, attached at the base of the calyx, this ca. 1.5 mm long; flower buds 1–1.5 × 0.5–1 mm, oval-elliptic. Flowers 1.5–2.5 cm long, papilionate; calyx 8.5–12.3 × 5.8–9.2 mm, densely ferruginous tomentose externally, internally green, the tube ca. 8.5–10 mm long, the lobes 5–7 × 3–4 mm, triangular, the adaxial pair partially joined; petals dark purple to black, free, glabrous, clawed at the base, the standard 9–16 × 6–9 mm, white stripes at the base, orbicular, deeply incised, basally rounded, apically emarginate, the wings 6.5–15 × 3.5–4.5 mm, oblong-lunate, the keel petals 6.5–15 × 2.5–3 mm, oblong-lunate, basally auriculate; stamens 10, in different sizes, the largest ca. 2 times larger than the smallest, the filaments of the smallest 4–6 mm long, the largest ones 12–14 mm long, free, glabrous, basally dilated, apically curved, anthers of the smallest stamens 1.0–1.5 × 0.2–0.4 mm, anthers of the largest ones 1.5–2.0 × 0.2–0.4 mm, basifixed, elliptic to oblong in outline; intrastaminal disc ring-shaped, glabrous, compressed; gynoecium 6–8 mm long, the ovary 5–6 × 1.2–2.0 mm, oblong in outline, laterally compressed, uniformly densely pilose, ovary subtended on a stipe 1–2 mm long, 3-ovulate, the style ca. 7 mm long, glabrous, apically curved, the stigma laterally bilobed. Fruit 2.5–7.5 × 1.4–2.5 cm, dehiscent along both sutures, suborbicular to ovate when one-seeded, oblong-elliptical when more than 2 seeds present, apically cuspidate, glabrous at maturity, the valves coriaceous to woody, 0.5–1.5 mm thick. Seeds 1–5, 9–11 × 8–10 mm, unicoloured, light to dark red, oval to suborbicular in outline, slightly compressed; hilum 2.3–2.8 × 1.3–1.6 mm, elliptic.

#### Additional specimens examined.

**Ecuador: Morona Santiago** • Limón Indanza, Cordillera del Cóndor, Centro Shuar Yunkuam, Cerro Chuank Naint (Vulture Mountain in Shuar language), collections made near a 1-hectare forest inventory plot, 17 Sep 2005, 1150 m a.s.l., 03°3'34"S, 78°14'45"W, *D.A. Neill & NSF dendrology course 14614* (MO, QCNE!). **Zamora Chinchipe** • cantón Nangaritza, Cordillera del Cóndor, tepui near Mirador del Nangaritza, directly northwest of ATASMO (Asociación de Trabajadores Autónomos San Miguel de las Orquídeas), drainage that includes río Chamico, 0.25 hectare tree inventory plot, “Rio Chamico” with the Lawrenceville School field course, 7 Mar 2017, 1353 m a.s.l., 04°12'31"S, 78°40'55"W, *J.L. Clark, J.A. Mayr & D.A. Neill 15159* (ECUAMZ); • same locality, 8 Mar 2017, *J.L. Clark, J.A. Mayr & D.A. Neill 15217* (ECUAMZ, G, MO, NY, SEL, US); cantón Nangaritza, parroquia Zumi, Cordillera del Cóndor, western side of tepui (bloque 2) that overlooks Río Nangaritza, directly east of Cabañas Yankuam, north of Reserva Natural Maycú and located in Área de Conservación ATASMO (Asociación de Trabajadores Autónomos San Miguel de las Orquídeas), 0–25-hectare plot with the Lawrenceville School field course, 1400 m a.s.l., 7 Mar 2018, 04°15'13.8"S, 78°38'11.6"W, *J.L. Clark & D.A. Neill 15612* (ECUAMZ, SEL); same locality, 7 Mar 2018, *J.L. Clark & D.A. Neill 15633* (ECUAMZ, MO, SEL, US); same locality, 7 Mar 2018, *J.L. Clark & D.A. Neill 15638* (ECUAMZ, F, G, MO, NY, SEL, US); same locality, 7 March 2018, *J.L. Clark & D.A. Neill 15649* (BM, CAS, ECUAMZ!, E, F, FLAS, G, MO, NY, SEL, US); • same locality, 7 March 2018, *J.L. Clark & D.A. Neill 15667* (ECUAMZ, MO, SEL, US); • same locality, 7 March 2018, *J.L. Clark & D.A. Neill 15677* (ECUAMZ, MO, SEL, US); • same locality, 7 March 2018, *J.L. Clark & D.A. Neill 15693* (ECUAMZ, F, G, SEL); • same locality, 7 March 2018, *J.L. Clark & D.A. Neill 15697* (ECUAMZ, MO, SEL); • same locality, 7 March 2018, *J.L. Clark & D.A. Neill 15715* (ECUAMZ, MO, SEL); • same locality, 8 March 2018, 04°15'13.8"S, 78°38'11.6"W, *J.L. Clark & D.A. Neill 15736* (ECUAMZ!, MO, SEL); • same locality, 8 March 2018, 04°15'13.8"S, 78°38'11.6"W, *J.L. Clark & D.A. Neill 15756* (ECUAMZ!, MO, SEL); • same locality, 8 March 2018, *J.L. Clark & D.A. Neill 15767* (ECUAMZ!, SEL!, US); • Cantón Nangaritza, Parroquia Zurmi, Cordillera del Cóndor, sloping sandstone tepui, east of Río Nangartiza, 2 km southeast of Las Orquídeas Village, in Área de Conservación de Las Orquídeas, 0.25-hectare forest inventory plot, “Parcela Atasmo Norte” with the Lawreenceville School field course, 7 Mar 2019, 1515 m, 4°14'14"S, 78°38'33"W. *J.L. Clark, D.A. Neill, E. Merino & A. Wilcox 16085* (ECUAMZ, LOJA, SEL); same locality, 7 Mar 2019, *J.L. Clark, D.A. Neill, E. Merino & A. Wilcox 16085* (ECUAMZ, SEL); • Cordillera del Cóndor Región, upper Río Nangaritza, “Area de Conservación los Tepuyes”, on upper portion of sloping sandstone plateau southwest of Las Orquídeas, near 1-hectare forest inventory plot “Nangaritza Upper Sandstone Plateau Plot”, 1620 m a.s.l., 6 Nov 2006, 04°15'13.8"S, 78°38'11.6"W, *D.A. Neill & NSF dendrology course 15465* (MO, QCNE, ECUAMZ); • Cordillera del Cóndor, Upper Nangaritza River, Comunidad Las Orquídeas, tepui east to Cabañas Yankuam in Reserva Natural Maycú, 1480 m a.s.l., 18 Oct 2024, 04°15'29.56"S, 78°38'19.59"W, *J.E Guevara, M.J. Endara & W. Raura 6790* (F, QCA, QCNE); • same locality, 18 Oct 2024, 04°15'29.56"S, 78°38'19.59"W. *J.E Guevara, M.J. Endara & W. Raura 6789* (F, QCA, QCNE).

#### Distribution and habitat.

**Figure 2.**
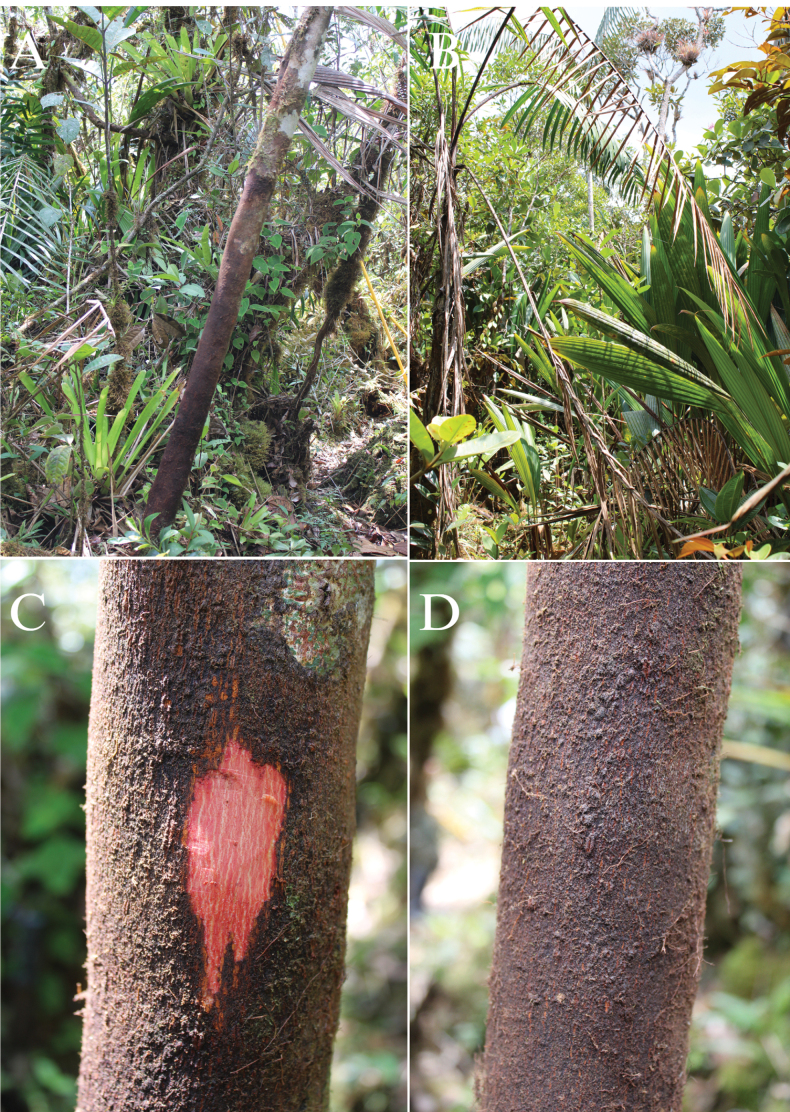
*Ormosianeillii* J.L. Clark & J.E. Guevara **A** habit **B** habitat **C** inner bark **D** outer bark. Photos by J.E. Guevara.

*Ormosianeillii* is a medium-sized tree to 15 m tall and only known from two localities on sandstone plateaus of the Cordillera del Cóndor (Clark and Neill 2023) (Figs 2, 5). It is locally abundant on white sand dwarf forests on Andean tepui-like formations above 1000 m altitude (Figs 2A, B, 5B). The maximum height observed in the field was 15 m and when cut, the inner bark is remarkably reddish (Fig. 2C, D). Some conspicuous floristic elements of this habitat include *Sterigmapetalumobovatum* Kuhlm. (Rhizophoraceae), *Humiriastrummapiriensis* Cuatrec. (Humiriaceae), *Andira* sp. nov. (Fabaceae), *Sloaneatiwintza* T.D. Penn. (Elaeocarpaceae), *Wettinialongipetala* A. Gentry (Araceae), *Psamnisia* sp. (Ericaceae), *Cybianthusmagnus* (Mez) Pipoly (Primulaceae) and *Ladenbergiafranciscana* C.M. Taylor (Rubiaceae). This area forms part of a landscape of isolated, tepui-like plateaus dating back to the Cretaceous period, characterised by low-stature forests rich in small trees with slender stems (Guevara and Fernández-Alonso 2018; Huamantupa-Chuquimaco and Neill 2018; Clark and Neill 2023). The upper soil layer consists of 20–50 cm of litter, beneath which soils rich in quartzitic white sands predominate. These types of environments in the Cordillera del Cóndor and other mountain ranges to the east of the Andean Mountain range in Ecuador and Peru are commonly referred to as “Andean tepuis” (Neill et al. 2014; Clark and Neill 2023). *Ormosianeillii* is endemic to Ecuador, but it is also expected to occur in similar sandstone habitats of unexplored regions of the Cordillera del Cóndor in Peru.

**Figure 3.**
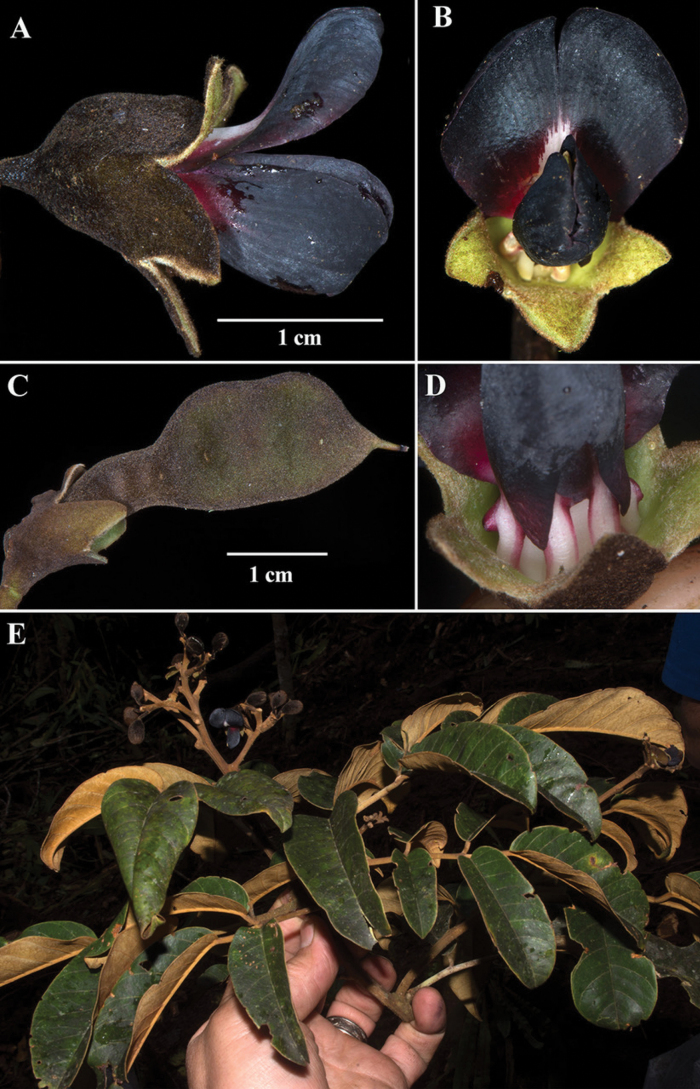
*Ormosianeillii* J.L. Clark & J.E. Guevara **A–D** flowers featuring remarkable dark purple to black corolla **E** flowering branch. Photos by John L. Clark of the type (J.L. Clark & D.A. Neill 15775).

**Figure 4.**
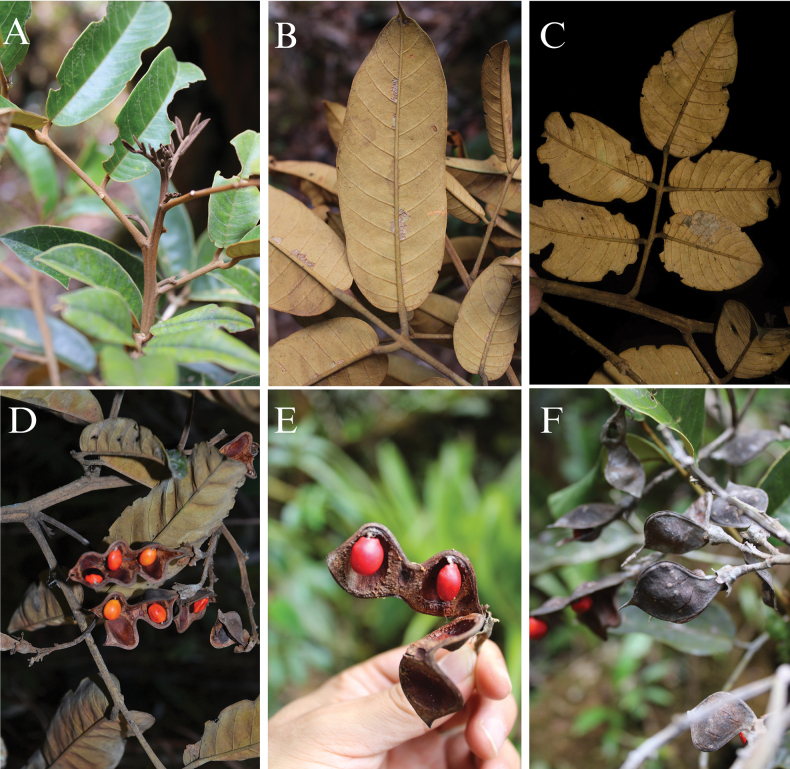
*Ormosianeillii* J.L. Clark & J.E. Guevara **A–C** details of the adaxial and abaxial surface of leaflets **D** fruiting branches with mature fruits **E–F** mature fruits showing details of sutures and cuspidate apex in the dehiscent fruit **A, B** from *J.E. Guevara et al. 6791* (QCNE, F) **C** from *J.L. Clark et. al. 16085***D** from *D.A. Neill et al. 17047***E–F** from *J.E. Guevara et al. 6790*. Photos **A, B, E, F** by J.E. Guevara **C, D** by J.L. Clark.

**Figure 5.**
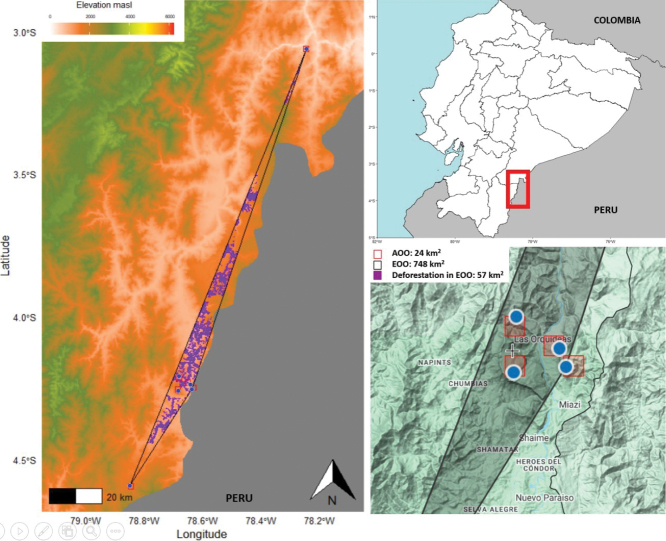
Conservation assessment for *Ormosianeillii* J.L. Clark & J.E. Guevara. The left panel shows the Extent of Occurrence (EOO) of *Ormosianeillii*, illustrating the current effects of deforestation across its known geographic range. The bottom right panel displays the Area of Occupancy (AOO) for *O.neillii*.

#### Etymology.

The specific epithet honours the botanical legacy of Dr David A. Neill (1953–2025), an American botanist who dedicated over three decades to the study of Ecuadorian flora. Dr Neill conducted extensive botanical surveys throughout Ecuador and played a pivotal role in mentoring numerous generations of botanists through his teaching, research and service. A passionate advocate for both botanical science and habitat conservation, he was instrumental in the establishment of several biological research stations in collaboration with the Jatun Sacha Foundation, a non-profit NGO he helped establish in the 1980s. His taxonomic expertise, particularly within the Fabaceae family, is widely acknowledged. This epithet serves as a fitting tribute to his legacy in plant systematics, Fabaceae taxonomy and his invaluable contributions to the field of botany.

#### Conservation status.

**Figure 6.**
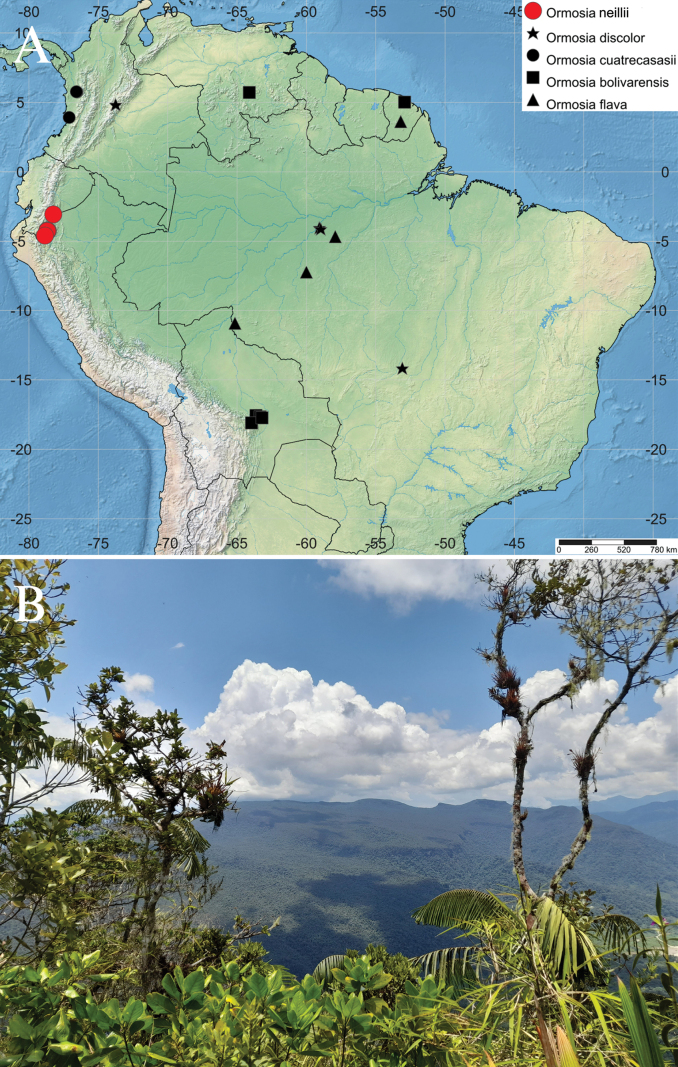
**A** Map of the geographic distribution of *Ormosianeillii* J.L. Clark & J.E. Guevara (red dots) and its morphologically most similar species **B** Cordillera del Cóndor landscape where *O.neillii* is frequent in the white sand dwarf forests on tepui-like formations. Photo by J. E. Guevara.

The range size analysis estimated an EOO of 748.8 km^2^ and an AOO of 24 km^2^. Our analysis also revealed that, since 2000, this species has suffered a significant reduction in its habitat quality considering a reduction of 7% for AOO and 11% for EOO. *Ormosianeillii* is only known from three localities in the Upper Nangaritza River and one locality in Cerro Plateado, all in the Cordillera del Cóndor Region (Fig. 6). Extensive clear-cutting during the last ten years has resulted in a drastic reduction of native forests (Tapia-Armijos et al. 2015). The expansion of both legal and illegal mining are additional major threats in the region (CEECEC [Civil Society Engagement with ECological EConomics] 2024). *Ormosianeillii* is preliminarily assessed as Endangered (EN), based on the following IUCN (2022) criteria: B1, B2ab (i,ii,iii) where EOO is less than 5,000 km^2^ and subcriteria indicate continuing decline, observed, inferred or projected, in area, extent and/or quality of habitat.

## ﻿Discussion

Following the classification proposed by Rudd (1965) and based on our description, *Ormosianeillii* is recognised in the sect. Ormosia because the sericeous pubescence on the abaxial surface of leaflets; secondary veins straight and almost parallel to the mid-vein; more than nine pairs of secondary veins; the dark-purple to black corolla and the glabrescent dehiscent fruit with ligneous or subligneous valves (Figs 3, 4D–F). The mostly unicoloured light red or dark red seeds of this species is a noteworthy characteristic (Fig. 4E–F). However, the above-mentioned characters present in this species are morphologically more similar to sect. Ormosia than to members of sect. Unicolores. The new species most closely resembles *O.cuatrecasasii* and *O.discolor*. Nonetheless, *O.neillii* is readily distinguished from *O.cuatrecasasii* by smaller leaves (5.5–14.5 cm long vs. 7–24 cm long), fewer secondary veins [10–12 vs. 11–14(–16)], longer calyx tube (10–15 mm long vs. 6–7 mm long), smaller fruits (3.5–6 cm long vs. 5–10 cm long), unicoloured seeds (vs. bicoloured red and black seeds), shorter hilum (1–1.5 mm long vs. 3 mm long) and fewer seeds per pod (1–3 vs. 1–6). A summary of these characters is provided in Table 1. The distribution of *O.neillii* in the eastern Andes of southern Ecuador is geographically isolated from *O.cuatrecasasii* in the Chocó Biogeographic Region in the western Andes of northern Ecuador and southern Colombia (Fig. 6)

**Table 1.** Diagnostic characters for *Ormosianeillii* and morphologically similar species, as well as their geographic distribution in the Neotropics: Western Amazon (WA), Central Amazon (CA) and the Guiana Shield (GS).

Characters	*Ormosianeillii*	*Ormosiadiscolor*	*Ormosiabolivarensis*	*Ormosiaflava*	*Ormosiacuatrecasasii*
Leaflet number	5–7	(3–)5–9	5–9	5–11	7–9
Leaflet size (cm)	5.5–14.5 × 3–6	7–30 × 4–12	10–18 × 5–15	4–14 × 2–6	7–24 × 4–11
Leaflet shape	Oblong-ellipitic	Oblong-ovate	Elliptic to oblong-elliptic	Elliptic to oblong-elliptic	Elliptic-ovate
Leaflet apex	Acute	Acuminate	Acute to breviacuminate	Acute to broadly acuminate	Acuminate
Leaflet base	Subcordate	Attenuate to slightly subcordate	Obtuse to subcordate	Obtuse	Obtuse to subcordate
Number of pairs of secondary veins	10–12	15–16	10–15	8–9	11–14
Stipules	Absent	Present	Present	Present	Absent
Flower length (mm)	15–25	6–8	15–20	15–18	NA
Corolla colour	Dark purple with inner white stripes	Black to blackish-purple	Dark purple	Yellow	NA
Calyx tube (mm)	10–15	4–6	5–10	6–10	6–7
Fruit size (cm)	3.5–6 × 2–3	2–5 × 1.5–2	3–8 × 2–3	3–5 × 1–2	5–10
Fruit apex	Cuspidate	Acute to strongly acuminate	Acuminate	Acuminate	Acute
Seed number	1–3	1–2	1–6	1–3	1–6
Seeds (mm)	10–10.3 × 8	9–11 × 8	8–11 × 7–10	10–14 × 9–14	10–11 × 9–10
Distribution range	WA	CA, GS	CA, GS	CA, GS	Chocó

*Ormosianeillii* is also distinguished from *O.discolor*, a morphologically similar species that inhabits terra firme forests in Central Amazonia. However, *O.neillii* can be readily differentiated from *O.discolor* in having larger fruits (3.5–6 × 2–3 cm long vs. 2–5 × 1.5–2 cm long) with strongly cuspidate apex (vs. acute to acuminate apex), larger flowers (15–25 mm long vs. 6–8 mm long) and longer calyx tube (10–15 mm long vs. 4–6 mm long). It also differs from *O.discolor* in having leaflets with fewer secondary veins (10–12 vs. 15–20) and fruits glabrescent in maturity (vs. fruits covered by minutely fulvo to ferruginous-velutinous pubescence).

In a recent molecular phylogenetic study, Torke et al. (2022) suggested the monophyly of the Nobilisoid clade may be congruent with Rudd’s (1965) classification corresponding to series *Nobiles* Rudd. The Nobilisoid clade is defined by variation in the colouring pattern of the seeds (Torke et al. 2022) and includes the following species: *Ormosianobilis*, *O.macrophylla*, *O.krugii* and *O.santaremensis*. This variation includes entirely red to bicoloured or entirely black seeds from single individuals or even the same pod (Rudd 1965). However, *O.neillii* shows a consistent pattern of monochromatic seed colour from light to dark red. In addition, the series *Nobilis* Rudd includes all the species morphologically similar to *O.neillii* and described in this study, but not all the species in this series were included in the molecular phylogenetic analysis of Torke et al. (2022). In the same study, the authors suggest that *O.discolor* is a divergent lineage within this clade and would constitute its sister taxon (Torke et al. 2022). Thus, there is uncertainty about the most likely placement of *O.neillii* in the *Ormosia* phylogeny, specifically within the Nobilisoid clade proposed by Torke et al. (2022).

It is also interesting to note that *O.cuatrecasasii*, the species that most closely resembles the new species, inhabits mostly humid forests of the Chocó Region in Colombia and no records from this species have been found in the eastern flanks of the Andes. Thus, despite the morphological similarities for both species, these forests have been geographically isolated for at least the last 10 Mya, which is the estimated divergence time for the Nobilisoid clade (Torke et al. 2022). However, a phylogenetic study including *O.neillii* is necessary to evaluate its sister-group relationship.

## Supplementary Material

XML Treatment for Ormosia neillii

## ﻿Additional information

### Author ORCIDs

Juan Ernesto Guevara-Andino https://orcid.org/0000-0002-5433-6218

John L. Clark https://orcid.org/0000-0002-1414-6380

Daniel Navas-Muñoz https://orcid.org/0009-0001-1761-9147

### Data availability

All of the data that support the findings of this study are available in the main text.
